# Effects of Hip Flexion on Knee Extension and Flexion Isokinetic Angle-Specific Torques and HQ-Ratios

**DOI:** 10.1186/s40798-021-00330-w

**Published:** 2021-06-12

**Authors:** Christian Baumgart, Eduard Kurz, Jürgen Freiwald, Matthias Wilhelm Hoppe

**Affiliations:** 1 Department of Movement and Training Science, University of Wuppertal, Fuhlrottstraße 10, 42119 Wuppertal, Germany; 2 Department of Orthopedic and Trauma Surgery, Martin-Luther-University Halle-Wittenberg, Ernst-Grube-Str. 40, 06097 Halle (Saale), Germany; 3 Institute of Movement and Training Science I, University of Leipzig, Jahnallee 59, 04109 Leipzig, Germany

**Keywords:** Eccentric, Concentric, Hamstrings, Quadriceps, Statistical parametric mapping (SPM)

## Abstract

**Background and Methods:**

During isokinetic knee strength testing, the knee flexion angles that correspond to the measured torque values are rarely considered. Additionally, the hip flexion angle during seated testing diverges from that in the majority of daily life and sporting activities. Limited information concerning the influence of hip angle, muscle contraction mode, and velocity on the isokinetic knee strength over the entire range of motion (ROM) is available.

Twenty recreational athletes (10 females, 10 males; 23.3 ± 3.2 years; 72.1 ± 16.5 kg; 1.78 ± 0.07 m) were tested for isokinetic knee flexion and extension at 10° and 90° hip flexion with the following conditions: (i) concentric at 60°/s, (ii) concentric at 180°/s, and (iii) eccentric at 60°/s. The effects of hip angle, contraction mode, and velocity on angle-specific torques and HQ-ratios as well as conventional parameters (peak torques, angles at peak torque, and HQ-ratios) were analyzed using statistical parametric mapping and parametric ANOVAs, respectively.

**Results:**

Generally, the angle-specific and conventional torques and HQ-ratios were lower in the extended hip compared to a flexed hip joint. Thereby, in comparison to the knee extension, the torque values decreased to a greater extent during knee flexion but not consistent over the entire ROM. The torque values were greater at the lower velocity and eccentric mode, but the influence of the velocity and contraction mode were lower at shorter and greater muscle lengths, respectively.

**Conclusions:**

Isokinetic knee strength is influenced by the hip flexion angle. Therefore, a seated position during testing and training is questionable, because the hip joint is rarely flexed at 90° during daily life and sporting activities. Maximum knee strength is lower in supine position, which should be considered for training and testing. The angle-specific effects cannot be mirrored by the conventional parameters. Therefore, angle-specific analyses are recommended to obtain supplemental information and consequently to improve knee strength testing.

## Key Points

- The angle-specific analysis of isokinetic torques during knee extension and flexion reveals effects according to the hip flexion, velocity, and contraction mode that were not captured by peak torque and angles at peak torques.
- The angle-specific effects provide the importance of the hip flexion angle, velocity, and contraction mode for the individualization of knee strength training and testing within performance, prevention, and rehabilitation programs.
- HQ-ratios are angle-specific and affected by the hip flexion and velocity. Therefore, the concept of restoring single HQ-ratios calculated from peak torques during rehabilitation is questionable.

## Background

Muscle strength and imbalance between quadriceps and hamstring muscles are discussed as risk factors for non-contact anterior cruciate ligament as well as hamstring injuries [1, 2]. Consequently, prevention and rehabilitation procedures focus on strengthening and balancing the muscles that surround the knee joint [3]. However, after return-to-sport, strength deficits and imbalances may persist and potentially cause further injuries [4–7]. Within these topics, the interaction between quadriceps and hamstring muscles and the contribution of mono- and bi-articular muscles remain complex and are not fully understood [8].

Isokinetic dynamometers are commonly used to evaluate knee muscle strength in athletes and patients. Frequently, quadriceps and hamstring strength is measured during concentric and/or eccentric muscle contractions in a sitting position at different velocities (e.g., 60°/s and 180°/s) [9, 10]. In previous studies, peak torque values for knee extension and flexion as well as hamstring/quadriceps (HQ) ratios were typically calculated [9, 11, 12]. It was shown that an extended hip joint has an impact on isokinetic results due to the potential influence on the stretch-tension relationship, relative contribution of active contractile components, and neuromuscular control of the muscle [10, 13–15]. Moreover, an extended hip joint was recommended for sport-specific testing (e.g., sprinting) [10, 13–15]. At greater hip flexion, knee extension and flexion peak torques during isokinetic eccentric and concentric testing at different velocities increased, while the HQ-ratio did not change [10, 13]. Thereby, one study found a more pronounced increase of the eccentric compared to the concentric peak torque [10]. Also, conflicting results exist regarding the effects of the movement velocity on the HQ-ratio [10, 13, 16, 17]. When taken together, the findings indicate that more research is needed.

However, all of the previous studies, which have applied a data reduction of continuous torque-time curves to single values (e.g., peak torque, HQ-ratio), potentially loss important information [18, 19]. To solve this problem, a statistical parametric mapping (SPM) procedure that enables an analysis of continuous signals is promising [18]. Recently, the SPM approach was applied in an isokinetic study, showing that this innovative statistical approach can reveal clinically meaningful information in patients after ACL reconstruction that was not shown by conventional calculated parameters [20].

A further drawback is that the majority of the isokinetic studies did not focus on the knee flexion angles that correspond to the measured torques. Therefore, such studies provide limited information about the muscle strength over the entire range of motion (ROM) [21]. Only a few studies that have investigated the influence of the hip flexion angle, velocity, or contraction mode on different knee strength values have considered the knee angles at the measured peak torques [16, 17, 20, 22]. Generally, peak torques generated by knee extensor and flexor muscles occur at different knee joint angles [16, 22]. Moreover, it was shown that the concentric peak torque shifted at higher velocities to a more extended knee joint during extension and to a more flexed one during flexion [16, 20], while no changes were found during eccentric contractions [16]. Consequently, angle-specific HQ-ratios are unrelated to the HQ-ratios typically calculated from peak torques [17, 20]. Taken overall, and irrespective of the knowledge from previous isokinetic analyses, an angle-specific approach could lead to a much better understanding of force production over the entire ROM and consequently of the strength capacities of the knee joint [20]. Such knowledge is helpful to design optimized strength training programs for performance, prevention, and rehabilitation procedures.

Therefore, the aim of the study was to investigate the effects of hip flexion on knee extension and flexion isokinetic angle-specific torques and HQ-ratios measured eccentrically and concentrically at two velocities. Based on existing studies, it was hypothesized that peak torques and HQ-ratios differ according to the hip flexion, contraction mode, and movement velocity. It was further expected that those torque differences vary according to the knee flexion angle.

## Methods

Twenty young healthy recreational athletes, who trained at least three or more times a week in their sport, were recruited (sex, 10 females and 10 males; age, 23.3 ± 3.2 years; mass, 72.1 ± 16.5 kg; height, 1.78 ± 0.07 m). Since the main focus of our study was methodological in its nature (angle-specific analyses), we did not distinguish between sexes. At the time of testing, all participants were free of acute illness and injuries. Additionally, none of them had an injury at the hip, knee, or ankle joint that required surgery. Prior to testing, all participants gave their written consent to the study protocol, which was approved by the Ethics Committee of the local university. The participants were instructed to refrain from physical activity 24h prior to the tests. Before the measurements, all participants were asked if they were able to perform maximal isokinetic knee strength testing. All tests were performed in accordance with the ethical standards of the Helsinki Declaration.

All female and male participants performed a 5-min warm-up on a treadmill at 8 or 10 km/h, respectively. Isokinetic knee flexion and extension torques (ROM 0–90°) were then measured using a standard device (Cybex NORM, Humac, CA, USA) without a separate familiarization session. The participants were randomly positioned and fixed by straps in both a supine and seated position with a hip flexion of 10° and 90°, respectively. At each position, the left leg was tested for maximum reciprocal knee extension and flexion in the following order: (i) concentric mode at 60°/s, (ii) concentric mode at 180°/s, and (iii) eccentric mode at 60°/s. A standardized 2-min rest was given between each trial. Before each trial, 3–5 submaximal repetitions (≤80%) were performed to familiarize participants with each test condition. Thereafter, 5 maximal repetitions were recorded. All tests were guided by the same investigator.

Isokinetic data were measured at 100 Hz and gravity-corrected using a spline interpolation of three isometric measurements at 0°, 30°, and 60° knee flexion. The torque values were normalized to body mass. The torque and velocity data were filtered by a recursive second-order digital low-pass Butterworth filter using a cut-off frequency of 1 Hz to allow for a reliable cutting of the repetitions. The repetitions were then separated applying a 0.1 Nm/kg torque threshold. Failed repetitions due to non-typical shapes (e.g., a shorter ROM) were removed. Hence, a total of 977 extension and flexion torque-angle curves were generated in steps of 1° using linear interpolation. Thereafter, the mean of all extension and flexion torque-angle curves was computed for each participant, hip flexion angle, contraction mode, and movement velocity. Additionally, these curves were smoothed by a fifth-order Savitzky-Golay filter with a frame size of 21 to avoid jumps based on the different ROMs of the trials. Afterwards, the angle-specific HQ-ratios (hamstring/quadriceps) for each testing mode were calculated. In all participants and conditions (velocity, contraction mode, and hip flexion), a ROM of 9° to 87° was present and therefore was used for the statistical evaluation of the torque-angle and HQ-ratio curves. Finally, the peak extension and flexion torques, knee angles at the peak torques, work data, and conventional HQ-ratios [23] were computed from all filtered repetitions included in the angle-specific analyses. As the work data gave no additional information to that of the peak torques and the mean correlation between both parameters was r = 0.95, we have decided to present only the peak torque data to reduce the number of findings for the readers.

### Statistical Analyses

The descriptive statistics of the parametric isokinetic values are reported as means and standard deviations. The parameters were checked for normal distribution using the Shapiro-Wilk test. Since all data were normally distributed, they were analyzed by two separate two-factor repeated measure ANOVAs using factors of “hip flexion x velocity” and “hip flexion x contraction mode.” Effect sizes were calculated by partial eta-squared (η_p_^2^), with values ≥ 0.01, ≥ 0.06, and ≥ 0.14 indicating small, moderate, and large effects, respectively [24]. Moreover, two two-factor (“hip flexion x velocity” and “hip flexion x contraction mode”) repeated measure ANOVAs of SPM were used to compare the angle-specific torques and HQ-ratios. The scalar output statistic SPM{F} was calculated for the ROM between 9 and 87°, which allowed for the identification of statistically significant different regions of the curves [25]. The normality assumption of SPM was implicitly checked with the agreement between parametric and non-parametric results [18]. All data analyses and statistical analyses were performed using R 3.3.2 [26]. The SPM analyses were implemented in Python using the open-source package spm1d (v. 0.4, www.spm1d.org). Statistical significance was set at *p* < 0.05.

## Results

**Fig. 1:**
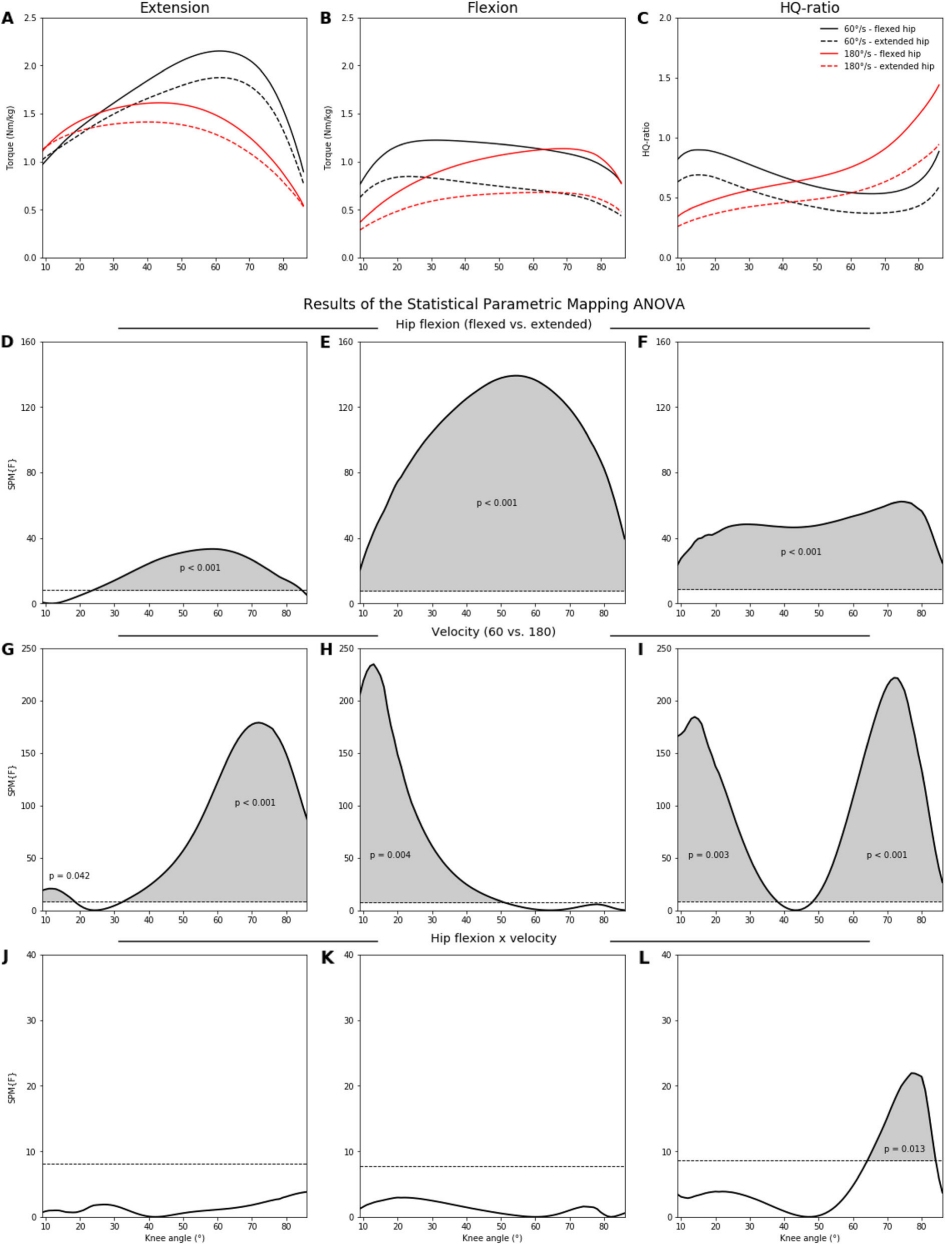
**A**–**C** Averaged angle-specific extension, flexion, and HQ-ratio curves separated for the flexed and extended hip joint as well as for the 60°/s and 180°/s velocity. **D**–**L** Results of the two-factor (hip flexion × velocity) repeated measure SPM ANOVAs. Gray shaded areas indicate the significant regions for each factor and their interaction. SPM statistical parametric mapping

**Fig. 2:**
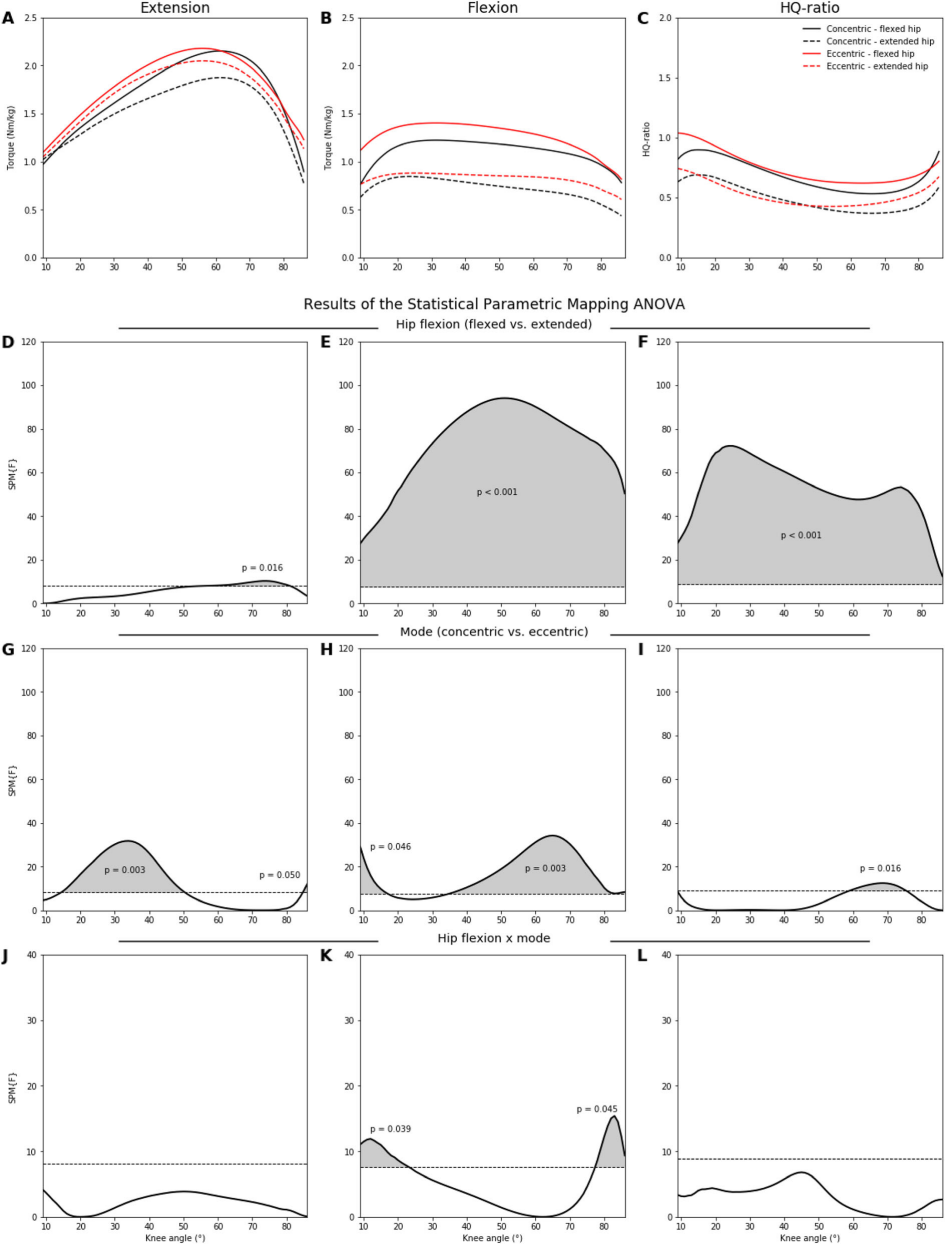
**A**–**C** Averaged angle-specific extension, flexion, and HQ-ratio curves separated for the flexed and extended hip joint as well as for the concentric and eccentric contraction mode. **D**–**L** Results of the two-factor (hip flexion × mode) repeated measure SPM ANOVAs. Gray shaded areas indicate the significant regions for each factor and their interaction. SPM statistical parametric mapping

**Fig. 3:**
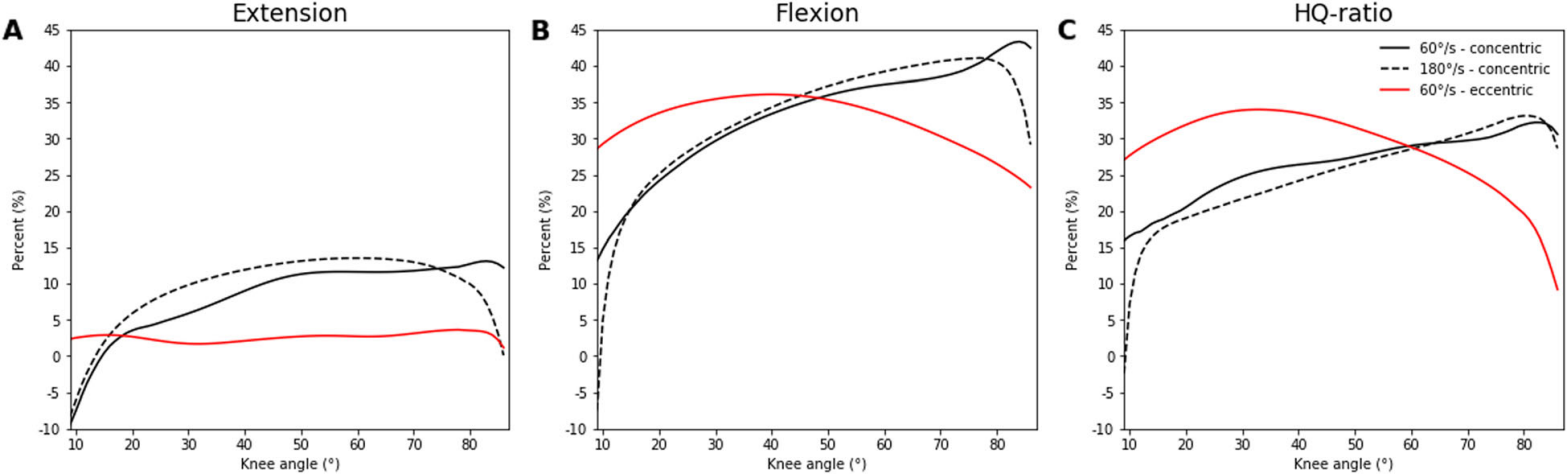
Percentage differences between the flexed and extended hip joint in angle-specific extension (**A**), flexion (**B**), and HQ-ratio (**C**) mean curves, separated for each testing mode

Table 1 summarizes all descriptive statistics and ANOVA outcomes of the peak extension and flexion torques with the corresponding knee angles and HQ-ratios. Figures 1 and 2 show the angle-specific extension, flexion, and HQ-ratio curves and also the results of the SPM ANOVAs. Figure 3 demonstrates the percentage differences between the angle-specific torques and HQ-ratios of the two hip flexion angles.

**Table 1:** Peak extension and flexion torques and corresponding knee angles as well as HQ-ratios as mean ± standard deviation

		Concentric				Eccentric		Statistics*					
		180°/s		60°/s		60°/s		ANOVA (hip flexion × velocity)			ANOVA (hip flexion × mode)		
		Hip flexed	Hip extended	Hip flexed	Hip extended	Hip flexed	Hip extended	Hip flexion	Velocity	Interact.	Hip flexion	Mode	Interact.
Extension	Torque (Nm/kg)	1.63 ± 0.20	1.44 ± 0.20	2.21 ± 0.43	1.95 ± 0.33	2.28 ± 0.55	2.18 ± 0.36	*p < .001* *η*_*p*_^*2*^ *= .62*	*p < .001* *η*_*p*_^*2*^ *= .84*	p = .305 *η*_*p*_^*2*^ *= .06*	*p = .016* *η*_*p*_^*2*^ *= .27*	*p = .009* *η*_*p*_^*2*^ *= .31*	p = .061 *η*_*p*_^*2*^ *= .17*
	Knee angle (°)	43.5 ± 7.6	38.9 ± 10.8	60.9 ± 6.9	59.6 ± 8.5	57.1 ± 9.8	57.0 ± 11.2	*p = .010* *η*_*p*_^*2*^ *= .30*	*p < .001* *η*_*p*_^*2*^ *= .94*	p = .077 *η*_*p*_^*2*^ *= .16*	p = .370 *η*_*p*_^*2*^ *= .04*	p = .155 *η*_*p*_^*2*^ *= .10*	p = .582 *η*_*p*_^*2*^ *= .02*
Flexion	Torque (Nm/kg)	1.16 ± 0.20	0.70 ± 0.18	1.26 ± 0.26	0.87 ± 0.18	1.47 ± 0.30	0.96 ± 0.17	*p < .001* *η*_*p*_^*2*^ *= .83*	*p < .001* *η*_*p*_^*2*^ *= .72*	p = .086 *η*_*p*_^*2*^ *= .15*	*p < .001* *η*_*p*_^*2*^ *= .80*	*p < .001* *η*_*p*_^*2*^ *= .49*	*p = .024* *η*_*p*_^*2*^ *= .24*
	Knee angle (°)	68.5 ± 6.9	61.2 ± 11.5	38.1 ± 13.2	26.9 ± 8.9	36.1 ± 15.2	32.4 ± 16.0	*p < .001* *η*_*p*_^*2*^ *= .63*	*p < .001* *η*_*p*_^*2*^ *= .91*	p = .299 *η*_*p*_^*2*^ *= .06*	*p = .001* *η*_*p*_^*2*^ *= .47*	p = .631 *η*_*p*_^*2*^ *= .01*	p = .058 *η*_*p*_^*2*^ *= .18*
HQ-ratio		0.71 ± 0.11	0.49 ± 0.13	0.58 ± 0.10	0.45 ± 0.08	0.66 ± 0.13	0.45 ± 0.09	*p < .001* *η*_*p*_^*2*^ *=.74*	*p < .001* *η*_*p*_^*2*^ *= .64*	*p < .001* *η*_*p*_^*2*^ *= .60*	*p < .001* *η*_*p*_^*2*^ *= .74*	*p = .018* *η*_*p*_^*2*^ *= .26*	*p = .001* *η*_*p*_^*2*^ *= .47*

Note: *two separate two-factor repeated measure ANOVAs

### Parametric Analyses (Hip Flexion × Velocity)

**Fig. 4:**
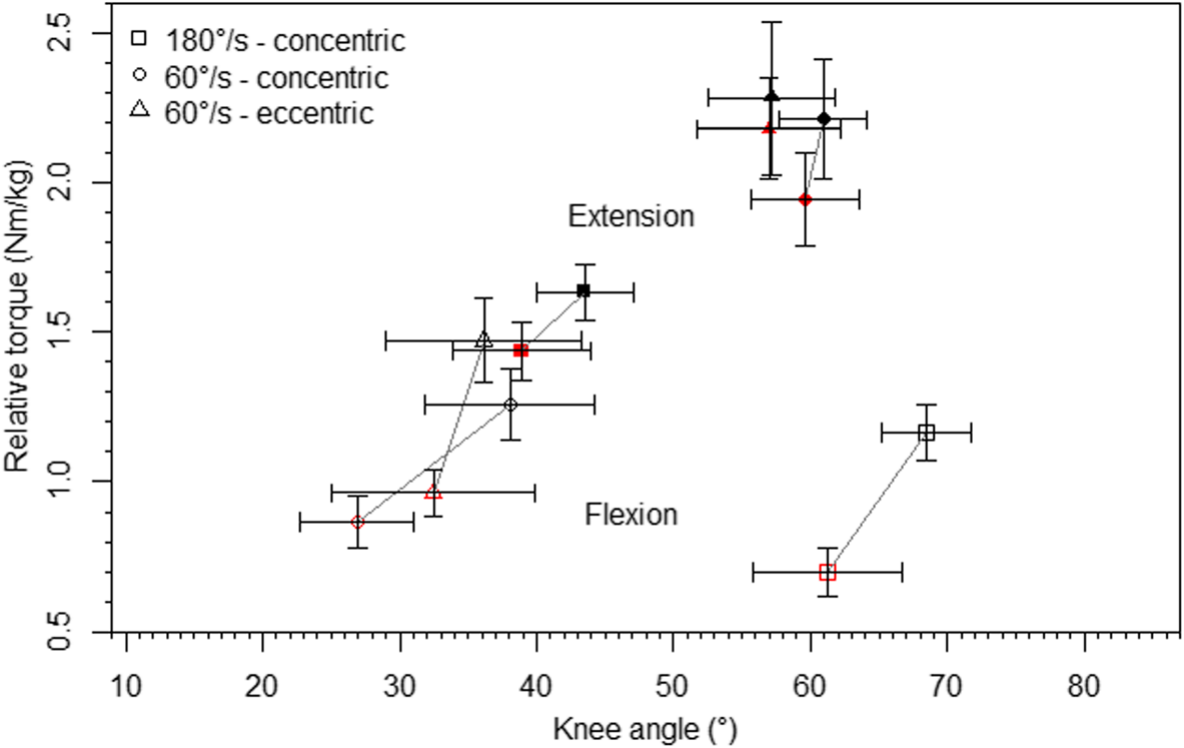
Peak extension (filled) and flexion (unfilled) torques and corresponding knee angles paired for both hip flexion angles (black—flexed hip, red—extended hip), displayed as mean and 95% confidence intervals

As shown by Table 1, the ANOVA revealed an interaction effect only for the HQ-ratios, while main effects for hip flexion and velocity were present in all parameters. Consequently, peak torques were higher at the flexed compared to the extended hip joint and were also higher at the slower compared to the higher velocity (Fig. 4). Knee flexion angles at peak torques were generally higher at the flexed hip. At the higher velocity, peak torques shifted towards a more extended and flexed knee angle during extension and flexion, respectively. Higher HQ-ratios were found at the higher velocity and with a flexed hip, while the difference between the HQ-ratios of both velocities was higher for the flexed hip.

### Parametric Analyses (Hip Flexion × Mode)

Presented also by Table 1, the ANOVA revealed an interaction effect for the knee flexion torques and HQ-ratios. Main effects for hip flexion were present in all parameters with the exception of the knee angle at the peak extension torque. Main effects of the contraction mode were found for the peak torques and HQ-ratio. Therefore, peak torques were higher at the flexed compared to the extended hip joint, and also higher during the eccentric mode. Thereby, the increase in the eccentric peak flexion torque was higher at the flexed hip joint. Compared to the concentric, knee angles at peak flexion torque were lower at the eccentric mode. Higher HQ-ratios were found at the eccentric mode and for the flexed hip, while the difference between both contraction modes was higher for the flexed hip.

### Angle-Specific Analysis (Hip Flexion × Velocity)

For extension and flexion torques and also HQ-ratios, main effects for the hip flexion were found over the entire ROM, with higher values at the flexed hip with the exception of 9 to 23° during extension. Main velocity effects were present in specific ROMs for extension (25 to 87°), flexion (9 to 50°), and HQ-ratios (9 to 37° and 48 to 87°). An interaction effect was found in the HQ-ratios from 64 to 85° knee flexion.

### Angle-Specific Analysis (Hip Flexion × Mode)

For flexion torques and HQ-ratios, main effects for hip flexion were found over the entire ROM, with higher values at the flexed hip. During extension, a main effect for hip flexion was found between 60 and 80° knee flexion. Main effects for contraction mode were present in specific ROMs for extension (15 to 5° and 85 to 86°), flexion (9 to 17° and 35 to 87°), and HQ-ratios (59 to 75°). An interaction effect was evident for the flexion torques from 9 to 22° and 78 to 87° knee flexion.

## Discussion

The aim of this study was to investigate the effects of hip flexion on knee extension and flexion isokinetic angle-specific torques and HQ-ratios measured eccentrically and concentrically at two velocities. Our main outcome was that the parametric and angle-specific extension and flexion torques, and also HQ-ratios, differ according to the hip flexion, velocity, and contraction mode. However, the angle-specific analyses revealed that some of these differences were not consistent over the entire ROM and consequently cannot be detected by conventional parameters.

Overall, the extension and flexion torques and HQ-ratios were lower at the extended than flexed hip joint. These observations are in line with those from several previous studies, which have found lower peak torques of quadriceps and hamstring muscles at more extended hip joint angles [10, 14, 15, 27]. These effects may particularly be explained by the well-known force-length muscle characteristics [28]. Moreover, the role of mono- and bi-articular muscles may also clarify some aspects [28]. We have found that testing with an extended hip joint decreases knee flexion torques to a greater extent than knee extension torques, which is in line with previous study findings [10, 15]. As only the rectus femoris muscle is a bi-articular quadriceps muscle, the extension strength is less affected by the hip flexion angle [29]. Moreover, the rectus femoris muscle has a lower impact on knee extension strength at low knee flexion angles. This is underpinned by a case study that has shown that the isokinetic extension torque was more affected at higher knee flexion after a total proximal rupture of the rectus femoris muscle [30]. In contrast, the hamstring muscles have an important role as hip extensors, and consequently, the influence of the hip flexion is much greater. From a macroscopic point of view, the hamstring muscle length at the flexed hip and knee joint is medium and comparable to that at the extended hip and knee joint. Therefore, almost completely different hamstring muscle lengths were tested at the two hip flexion angles [31]. Moreover, at the medium hamstring muscle length (flexed hip and knee joint as well as extended hip and knee joint), the differences between eccentric and concentric contractions were negligible (see Fig. 2B). Contrastingly, the highest hamstring strength was observed eccentrically at long muscle lengths (flexed hip and extended knee joint). This corresponds to the late swing phase during sprinting, where most hamstring strains occur, and which was effectively addressed by eccentric preventive training procedures in previous studies [32]. Whether the amount of the influence of hip flexion on the knee extension and flexion strength differs between different groups and/or could be changed (e.g., by training) remains unknown and should be addressed by more research.

However, and for the first time, we showed that the influence of the two hip flexion angles on angle-specific torques and HQ-ratios are not consistent over the entire ROM (see Figs. 1, 2, and 3). While the amounts and contours of the percentage differences between the two hip flexion angles are comparable for both concentric velocities, those for the eccentric contraction mode are different (see Fig. 3). During the concentric extensions, 5 to 12% lower torques were present at a knee flexion ROM of 20 to 75°. The torques of the eccentric extensions, in contrast, were less affected by hip flexion. Therefore, the mono-articular portions of the quadriceps were potentially more important during single joint eccentric contractions than the bi-articular rectus femoris muscle. The hip flexion angle influenced the knee flexion torques considerably more, but this influence was not constant over the entire ROM. For the concentric contractions, the percentage differences increased from 15 to 40% at higher knee flexion, while they oscillated only between 25 and 30% during the eccentric contraction mode (see Fig. 3). Therefore, the shortening of the functional length of the bi-articular hamstrings muscles has a greater impact on the concentric than the eccentric strength. With this in mind, the role of titin within the three filament theory may also be an explanatory point [33]. In our study, during the eccentric contractions, the differences between both hip flexion angles were almost constant over the entire knee ROM. Speculatively, the initial muscle fiber lengths have a low impact on the knee ROM-induced changes for the eccentric strength, which may be explained by the ability of titin to change its stiffness and length [33]. The shapes of the percentage difference curves in the HQ-ratios between the two hip flexion angles were almost identical to those found for the flexion torques. A decrease in hip flexion generally decreases the HQ-ratio over the entire ROM regardless of velocity and contraction mode. This was partly shown for selected knee flexion angles and concentric contractions before [17]. In contrast to our results, no differences in HQ-ratios calculated from peak torques were reported between different hip flexion angles from 0 to 120° [10, 13].

Supported by previous studies [15, 16], we found a general decrease in peak torques at higher velocities during knee extension and flexion. However, at the higher velocity, the knee flexion angles at the peak torque decreased during knee extension but increased during knee flexion (see Fig. 4). Thus, the angle-specific torques differ between both velocities, although not over the entire ROM. Irrespective of the hip flexion angle, no differences between 60 and 180°/s were revealed during knee extension and flexion in knee flexion angles of <30° and >50°, respectively. Therefore, at shorter muscle lengths, the velocity has no influence on the concentric strength output of the quadriceps and hamstring muscles. Consequently, the angle-specific HQ-ratios were also influenced by the velocity. In knee flexion angles <43°, the HQ-ratios at 180°/s were lower than those at 60°/s, but higher in knee flexion angles >43°. Interestingly, this reversal occurred at the same knee flexion angle irrespective of the hip flexion. Therefore, the influence of the movement velocity on the angle-specific HQ-ratios was comparable between both hip flexion angles regardless of the different muscle lengths. Thus, the need for angle-specific analyses in isokinetic studies was also underlined by these results.

Our outcomes further show that the knee angles at peak torque were not influenced by the contraction mode (Fig. 4). Higher peak torques were found during the eccentric compared to the concentric mode, which was also shown before [16]. However, the angle-specific torques revealed that the effects of the contraction mode were not consistent over the entire ROM. During knee extension and flexion, significant greater eccentric torque values were localized at lower and higher knee flexion angles, respectively. Thereby, higher HQ-ratios were found during the eccentric contraction, but only between 60 and 75° knee flexion.

### Practical Applications

The quadriceps and hamstring strength during isokinetic knee testing is influenced by the hip flexion. From this perspective, existing isokinetic testing and training exercises are questionable, because during daily life and sporting activities the hip joint is rarely flexed at 90°. Moreover, the hamstring and quadriceps strength is considerably higher during sitting. Thus, when biological structures need to be loaded cautiously (e.g., during early rehabilitation phases), an extended hip joint may be favorable to potentially reduce the absolute load of selected tissue (e.g., retro-patellar cartilage). Moreover, this study shows that parametric peak torques and HQ-ratios did not allow an evaluation over the entire ROM. Therefore, guiding rehabilitation by HQ-ratios based on peak torques is questionable.

### Limitations

A few limitations of the study have to be acknowledged. First, only the left leg was tested. The reason for this was to reduce the volume of exercises for each participant and to avoid possible fatigue effects. Second, we were unable to measure the muscle activity. Thus, we can only speculate regarding the potential effects of the hip flexion on the contribution of synergistic or antagonistic muscles. A further point is that we have only calculated conventional and angle-specific HQ-ratios. The reason for excluding other HQ-ratios was their low evidence as sensitive clinical tools for predicting injuries or monitoring knee joint integrity [12]. We have not considered potential deviations of the movement data measured by the dynamometer as well as the acceleration and deceleration of the lever arm [34]. Therefore, our data analyses may include potential inertia effects. However, regarding our results, the size of the potential affected ROM is negligible [20, 35]. Lastly, isokinetic knee testing is a mono-articular movement and not similar to most of the movements in daily life and sporting activities, which involve variable velocities and multiple joints. Therein, bi-articular muscles often work quasi-isometrically, while mono-articular muscles were shorten and lengthen [28]. This aspect could not be considered using isokinetic devices.

## Conclusions

Our study shows that parametric and angle-specific extension and flexion torques, and also HQ-ratios, differ according to the hip flexion, velocity, and muscle contraction mode. However, the angle-specific analyses revealed that some of these differences were not consistent over the entire ROM and consequently cannot be detected by conventional parameters. Based on our results, the use of an angle-specific analysis is recommended in future studies to facilitate strength performance at different muscle lengths. The influence of hip flexion, velocity, and contraction mode should be considered for more individualized isokinetic testing routines within strength performance, prevention, and rehabilitation programs.

## Data Availability

The datasets of the current study are available from the corresponding author on reasonable request.

## Abbreviations

ACL: Anterior cruciate ligament
HQ: Hamstring to quadriceps
ROM: Range of motion
SPM: Statistical parametric mapping
